# Digital escapism amid conflict: media and gaming behaviors in the shadow of war

**DOI:** 10.17179/excli2025-8592

**Published:** 2025-07-10

**Authors:** Muddsar Hameed

**Affiliations:** 1 College of Education, Psychology and Social Work, Flinders University, Sturt Rd, Bedford Park Adelaide SA 5042, Adelaide, Australia

## ⁯

Recent flare-ups in geopolitical tensions between India and Pakistan have rekindled old insecurities over regional instability and evoked intense public interest in both countries. Although not all civilians participate directly in armed conflict, the psychological pressure of such hostilities does not reach the end of the battle lines alone. Regular exposure to the media with a violent backdrop, and uncertainty about the country's security augmented by the politicization of social narratives leads to a sense of collective stress, anxiety, and emotional fatigue among the population, including particularly susceptible groups of youth and young people (Masood, 2020[6]).

Several past studies on the effects of war in conflict areas have reported the lesser, yet widespread impact of war on civilian mental health, showing an increase in posttraumatic stress symptoms, sleep disorders, and emotional dysregulation in non-combatants. In particular, the younger population, who are both hyperconnected and developmentally inclined toward social disruption, usually escape to the digital space where they sometimes seek to process or disassociate from the overstretched social realities (Lim, 2024[5]). This can trap individuals in algorithmic newsfeeds and emotionally charged content, heightening distress instead of alleviating it.

### Addictive media consumption during crisis

Digital media platforms serve as sources of knowledge and communication. While they offer real-time updates and social solidarity, they also foster compulsive usage patterns excessive scrolling, repetitive news consumption, and emotional overinvestment known as media addiction (Vannucci et al., 2017[10]).

During unstable times, such as military escalation, exposure to negative news in social media often manifests as “doomscrolling” habitual searching for despair-inducing information. Consumption of misinformation or emotionally intense content on platforms like Twitter, TikTok, or WhatsApp may lead to vicarios trauma internalizing others' distress without direct exposure (Tandoc et al., 2015[9]). In South Asia, where media is polarized and digital penetration is rising, such dynamics complicate socio-emotional realities during crises.

### Gaming as a coping strategy

Despite sociopolitical turmoil, many adolescents and young adults turn to online games and virtual platforms not merely as entertainment but as psychologically immersive spaces offering relief from real-world stressors (Király et al., 2017[3]). During national crises, gaming becomes a self-soothing mechanism, restoring control, agency, and social connection. For example, MMORPGs (massively multiplayer online role-playing games) allow identity creation, goal-oriented activity, and virtual social support (Granic et al., 2014[1]).

In regions like the Middle East or Eastern Europe, researchers report that war trauma and displacement-related grief are often managed through digital gaming escapes (Hassoun et al., 2025[2]). While gaming supports resilience, it also risks overuse or dependency when it becomes a sole coping tool.

### Problematic vs. adaptive use

Gaming and media use can support emotional regulation, distraction, and social bonding but when prolonged or uncontrolled, they shift from adaptive to problematic. Adaptive use is typically short-term and mood-enhancing, while problematic use involves compulsivity, impaired control, and interference with social and occupational functioning resulting in isolation, academic decline, and sleep issues (Pontes and Griffiths, 2015[8]).

Furthermore, excessive crisis-related media use exacerbates rather than soothes distress, especially when exposed to triggering content, misinformation, or conflict propaganda (LaRose et al., 2010[4]). The distinction between coping and compulsion requires behavioral insight and individualized understanding.

### Call for mental health awareness and research

Amid national conflict, scholars, clinicians, and policymakers must integrate digital coping behaviors into post-conflict mental health paradigms. Youth engagement with technology now shapes psychosocial realities, necessitating evidence-informed frameworks of digital vulnerability and resilience. Urgent interdisciplinary research is needed on how media exposure, conflict, and behavioral addiction interrelate.

Such studies must assess not only psychological harm but also protective effects of gaming/media in trauma contexts, guiding culturally responsive, trauma-informed interventions (Patel et al., 2018[7]). Particularly in politically fragile and underserved regions like South Asia, integrating digital behavior screening into trauma protocols can enable early detection and targeted public health messaging.

In conclusion, future generations must be equipped with adaptive coping strategies through collaborative, interdisciplinary action balancing resilience and risk in the digital age.

## Conflict of interest

The author declares no financial or other conflicts of interest.
